# Regulatory Effects of Source–Sink Manipulations on Photosynthesis in Wheat with Different Source–Sink Relationships

**DOI:** 10.3390/plants14101456

**Published:** 2025-05-13

**Authors:** Siqi Zhang, Huimin Chai, Jiawei Sun, Yafang Zhang, Yanhua Lu, Dong Jiang, Tingbo Dai, Zhongwei Tian

**Affiliations:** 1 Institute of Agricultural Information Technology, Henan Academy of Agricultural Sciences, Zhengzhou 450008, China; 2019201025@njau.edu.cn; 2 Key Laboratory of Huang-Huai-Hai Smart Agricultural Technology, Ministry of Agriculture and Rural Areas, Zhengzhou 450008, China; 3 Key Laboratory of Crop Physiology Ecology and Production Management, Ministry of Agriculture/College of Agriculture, Nanjing Agricultural University, Nanjing 210095, China

**Keywords:** photosynthesis, source–sink manipulation, source–sink relationships, sink–source ratio, wheat

## Abstract

Understanding the contributions of source–sink relationships to photosynthesis will help achieve high wheat grain yields. A single-factor field experiment was conducted to quantify the regulatory effects of different sink–source ratios on wheat photosynthetic characteristics, including two wheat cultivars with different source–sink relationships as materials for detailed source–sink manipulations through flag leaf removal (LR) and removal of spikelets on one side of each spike (SR). Compared with a control (CK), LR increased the sink–source ratio (23.84%) and significantly reduced the yield (16.17%), 1000-kernel weight (11.73%), and kernels per spike (7.33%). LR increased the leaves’ net photosynthetic rate (Pn) (4.27–15.82%), the electron transfer rate (3.97–14.93%), and the Rubisco activity (2.16–12.25%) in the short term, and LR increased sucrose synthesis-related enzyme activities (3.96–19.95%) and gene expressions *(SPS1*, *SUS1*, *CIN1*, and *SUT1*). Compared with CK, SR reduced the sink–source ratio (44.12%) and significantly increased the 1000-kernel weight (10.02%) but reduced the yield (43.93%) and kernels per spike (49.31%). SR reduced the leaves’ Pn (8.54–21.41%), the electron transfer rate (3.51–16.71%), and the Rubisco activity (5.96–21.51%), and the photosynthetic process was limited. SR decreased sucrose synthesis-related enzyme activities (5.12–29.09%) and gene expressions *(SPS1*, *SUS1*, *CIN1*, and *SUT1*). Therefore, a higher sink–source ratio is an important indicator of high photosynthetic efficiency, which can be used as a screening and judgment index in variety selection and cultivation regulation.

## 1. Introduction

Wheat is one of the most important and widespread food crops in the world, and it will be necessary to significantly increase its food production to meet the growing world population, which is expected to reach more than 9 billion by 2050 [1]. Wheat production will face severe constraints and challenges due to population growth and an increased demand for food, as well as the effects of unfavorable climate change [2]. After the source–sink theory was proposed in Mason and Maskel [3], several scholars studied the source–sink relationships of crops. In the absence of biotic or abiotic stresses, wheat grain yields depend heavily on the high levels of coordination among source activity, sink strength, and flow fluency [4]. Source–sink interactions regulate carbon assimilation and allocation, and an adequate supply of carbohydrates can accelerate the growth of sink organs and the formation of new sinks [5]. A wheat yield could be either increased by source activity and/or sink strength, or it can be limited by the smaller of these two components [6,7]. The key to achieving high crop yields is to optimize source–sink relationships [8]. A limitation of grain growth occurs when grain growth is limited by the grain sink size of earlier stages and by the photosynthetic capacity of later stages [5]. Quantifying the limitations of the source (primarily photosynthesis) and sink (demand for grain growth) to crop growth will provide a new understanding of improving wheat grain yields [9].

In the analysis of crop source–sink relationships, the sink–source ratio is commonly used as an important indicator of source–sink structures. A higher sink–source ratio is one of the main quality characteristics of high-light-efficient crop populations [10]. Yield improvement should be controlled in a suitable range, according to the sink–source ratio. It has been shown that a higher sink–source ratio can increase the net photosynthetic rate (Pn) and delay leaf senescence [11]. At the same time, the sink–source ratio is an important factor affecting assimilate partitioning. A high or low sink–source ratio will cause an imbalance between the supply and demand of assimilates. This suggests that there is a range of sink–source ratios, the magnitude of which largely determines the effectiveness of assimilates. The most common method utilized in the study of source–sink relationships is to artificially alter the sink–source ratio through source or sink removal treatments. Typically, the source manipulation is leaf removal or shading, while the sink manipulation is spike removal (e.g., pruning part of the spikelets), which determines the reduction or increase in source–sink relationships, respectively [12]. Source–sink manipulation experiments allow for estimation of the degree of limitation of post-flowering sources on grain yields of wheat cultivars [13,14]. Reynolds [15] found that wheat yields and biomasses were mainly sink-limited. A case of source–sink co-limitation has also been reported in wheat [16]. It is now generally recognized that the sink of modern wheat cultivars still contributes to yield increases, but there has been a shift through breeding from sink limitations to source limitations, and its source is becoming a limiting factor for further yield increases in the future. Therefore, breeders must focus more on increasing the pre- and post-anthesis photosynthetic capacity, while increasing the source size (e.g., post-anthesis photosynthetic capacity) and the sink size (number of grains per m^2^ and potential grain weight) are key tasks in the selection of modern wheat cultivars for high yield potential.

Many source–sink manipulations, such as pruning, leaf removal, flower thinning, and spikelet pruning, can affect alterations between the source and sink, resulting in a reduction or accumulation of leaf end-products, which in turn affects photosynthesis and the transport and partitioning of photosynthesis products between the source and sink organs [6]. Within a certain range, the larger the leaf area, the more photosynthetic leaf products there are. A better understanding of the contributions of plant organs in different source–sink relationships to the post-anthesis photosynthetic capacity is essential for overcoming source limitations during the late grain-filling period [17]. Grain growth responses to source–sink manipulation treatments that reduce the source size must take into account potential compensations for remaining photosynthetic organs. Studies have shown that appropriate removal of leaves improves light transmission performance in crop populations and that the compensatory effect of remaining leaves contributes to enhanced photosynthesis [18]. However, an excessive reduction in the source will not only make the leaf population’s photosynthesis and photosynthesis products decline but will reduce the yield [19]. Several studies show that enhancing the sink strength during the grain-filling period can improve photosynthesis and radiation-use efficiency [20,21]. It has also been confirmed that the accumulation of photosynthetic assimilates in leaves at low sink strengths is responsible for a decrease in the net photosynthetic rate [22]. Studies have shown that the removal of spikelets has significant inhibitory or reducing effects on the net photosynthetic rate, stomatal conductance, and intercellular CO_2_ concentration in flag leaves [11]. Therefore, optimizing source–sink relationships will provide a solution for realizing full yield potentials, so that the yield matches the photosynthetic potential of current and future genotypes. This will help in identifying lines with favorable source–sink relationships that can be used in physiological strategic crosses to increase potential yields.

In this study, we selected two wheat cultivars with source–sink relationships as test materials for detailed source–sink manipulation treatments (leaf removal and spikelet removal). Changes in the photosynthetic electron transfer capacity, Rubisco carboxylation capacity, and sucrose transport capacity of functional leaves in the source–sink manipulation treatments were estimated. The objectives were to quantify variations in the regulatory effects of different sink–source ratios on photosynthetic characteristics in wheat. This study aimed to determine the responsiveness of the source to future changes in the sink capacity to deepen our understanding of the regulatory effects of changes in source–sink relationships on source leaf photosynthesis and to provide a theoretical basis for the high-yield cultivation of wheat.

## 2. Results

### 2.1. Yield, Its Components, and Sink–Source Ratio

The results of the combined ANOVA analyses of yield-related components and sink–source ratios are shown in Table 1. YM25 had a significantly higher yield, kernels per spike, and 1000-kernel weight than YM1. Compared to control (CK), flag leaves removal (LR) significantly decreased the yield of YM25 and YM1 by 14.73% and 20.74% during 2021–2022 and by 13.13% and 19.05% during 2022–2023. LR significantly decreased the kernels per spike of YM25 and YM1 by 4.47% and 8.22% during 2021–2022 and by 7.46% and 9.17% during 2022–2023. LR significantly decreased the 1000-kernel weight of YM25 and YM1 by 12.58% and 15.44% during 2021–2022 and by 7.25% and 11.66% during 2022–2023. LR significantly increased the sink–source ratio of YM25 and YM1 by 26.75% and 17.09% during 2021–2022 and by 32.00% and 19.53% during 2022–2023.

Compared to CK, removal of spikelets on one side of each spike (SR) significantly decreased the yield of YM25 and YM1 by 40.28% and 47.67% during 2021–2022 and by 41.24% and 46.55% during 2022–2023. SR significantly decreased the kernels per spike of YM25 and YM1 by 46.42% and 51.29% during 2021–2022 and by 49.83% and 49.70% during 2022–2023. SR significantly increased the 1000-kernel weight of YM25 and YM1 by 11.75% and 7.99% during 2021–2022 and by 14.52% and 6.52% during 2022–2023. SR significantly decreased the sink–source ratio of YM25 and YM1 by 41.35% and 48.39% during 2021–2022 and by 40.55% and 46.20% during 2022–2023.

### 2.2. Chlorophyll SPAD

**Figure 1:**
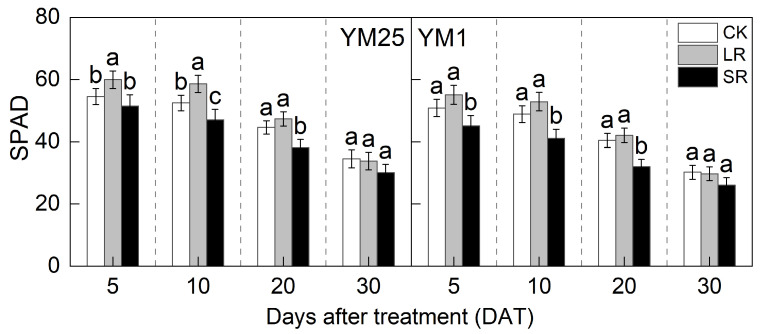
Effects of different source–sink manipulations on SPAD. CK, control; LR, flag leaf removal; SR, removal of spikelets on one side of each spike. Each value represents the mean ± SD of three replicates. Different lowercase letters indicate statistically significant differences between treatments (*p* < 0.05), according to the LSD test. Error bars indicate SDs.

The chlorophyll SPAD decreased with the number of days after treatment (Figure 1). For the LR treatment, the SPAD of YM25 was significantly higher than CK at 5 and 10 DATs. Compared to CK, LR increased SPAD in YM25 and YM1 by 6.10–11.67% and 4.05–8.30%, respectively. The above results indicate that a higher sink–source ratio promoted chlorophyll synthesis.

For the SR treatment, the SPAD of both cultivars was significantly lower than CK at 10 and 20 DATs. Compared to CK, SR decreased SPAD in YM25 and YM1 by 5.50–14.64% and 11.28–20.86%, respectively. The above results further confirm the adverse effect of low sink–source ratios on chlorophyll stability.

### 2.3. Pn and Photosynthetic Fluorescence Parameters

**Figure 2:**
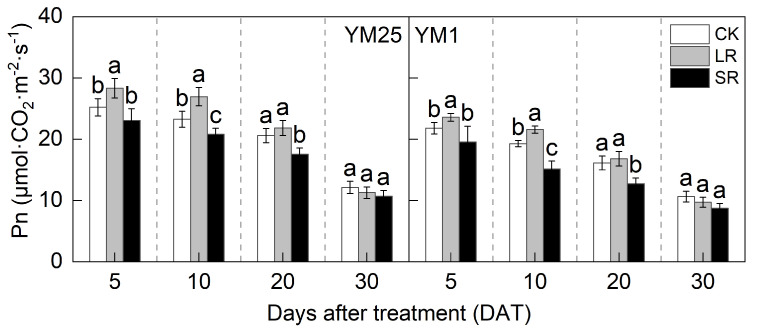
Effects of different source–sink manipulations on net photosynthetic rate (Pn). CK, control; LR, flag leaf removal; SR, removal of spikelets on one side of each spike. Each value represents the mean ± SD of three replicates. Different lowercase letters indicate statistically significant differences between treatments (*p* < 0.05), according to the LSD test. Error bars indicate SDs.

The Pn decreased with the number of days after treatment (Figure 2). For LR, the Pn of both cultivars was significantly higher at 5 and 10 DATs than that of CK. Compared to CK, LR significantly increased the Pn of YM25 and YM1 by 5.94–15.82% and 4.27–11.93%. For SR, the Pn of both cultivars was significantly lower at 10 and 20 DATs than that of CK. Compared to CK, SR significantly decreased the Pn of YM25 and YM1 by 8.54–14.86% and 10.25–21.41%.

**Figure 3:**
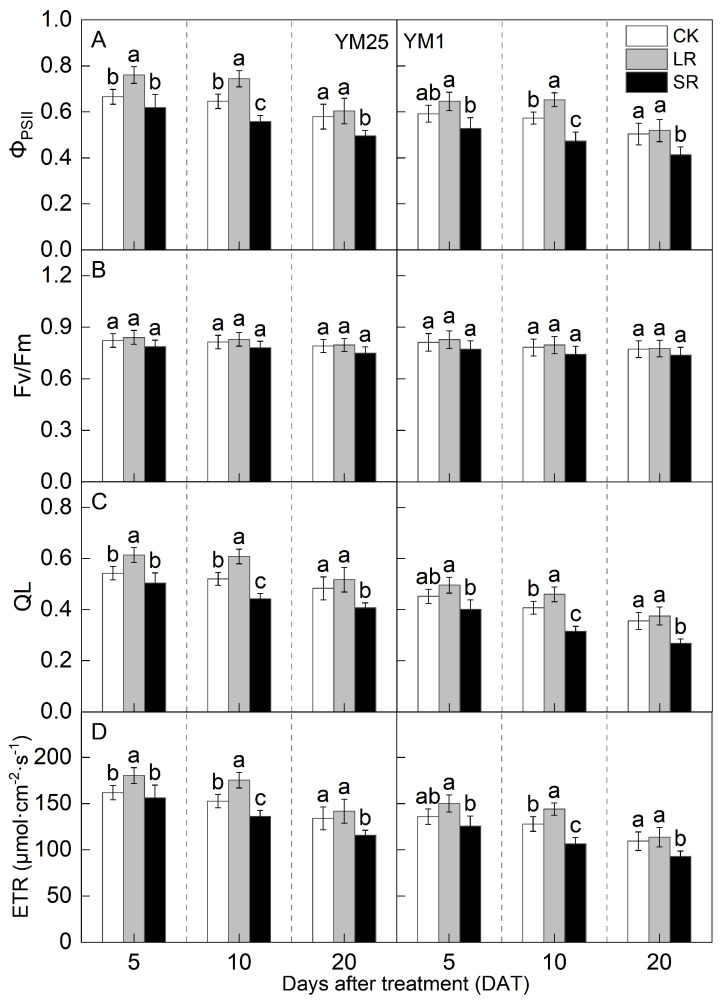
Effects of different source–sink manipulations on the actual quantum efficiency of PSII (Φ_PSII_) (**A**), the maximum quantum efficiency of PSII (F_v_/F_m_) (**B**), photochemical quenching (QL) (**C**), and the electron transport rate (ETR) (**D**). CK, control; LR, flag leaf removal; SR, removal of spikelets on one side of each spike. Each value represents the mean ± SD of three replicates. Different lowercase letters indicate statistically significant differences between treatments (*p* < 0.05), according to the LSD test. Error bars indicate SDs.

The values of the actual quantum efficiency of PSII (Φ_PSII_), photochemical quenching (QL), and the electron transport rate (ETR) decreased with the number of days after treatment (Figure 3A,C,D). There was no significant difference in the maximum quantum efficiency of PSII (F_v_/F_m_) (Figure 3B). For LR, the values of Φ_PSII_, QL, and ETR in YM25 were significantly higher at 5 and 10 DATs than those of CK. The values of Φ_PSII_, QL, and ETR in YM1 were significantly higher at 10 DATs than those of CK. Compared to CK, LR significantly increased the Φ_PSII_ of YM25 and YM1 by 4.34–15.22% and 2.98–13.95%. LR significantly increased the QL of YM25 and YM1 by 7.10–16.79% and 5.24–12.77%. LR significantly increased the ETR of YM25 and YM1 by 5.81–14.93% and 3.97–12.62%. The above results indicate that LR can increase the degree of openness of PSII, the actual photochemical efficiency, and the electron transfer capacity of remaining leaves.

For SR, the values of Φ_PSII_, QL, and ETR of both cultivars were significantly lower at 10 and 20 DATs than those of CK. Compared to CK, SR significantly decreased the Φ_PSII_ of YM25 and YM1 by 7.12–14.42% and 10.85–17.86%. SR significantly decreased the QL of YM25 and YM1 by 6.97–15.71% and 11.01–24.85%. SR significantly decreased the ETR of YM25 and YM1 by 3.51–13.57% and 7.64–16.71%. The above results indicate that SR can reduce the degree of openness of PSII, the actual photochemical efficiency, and the electron transfer capacity in leaves.

### 2.4. Carboxylation Efficiency (CE), Maximum Carboxylation Rate (V_cmax_), Maximum Electron Transport Rate (J_max_) and Triose Phosphate Utilization Rate (V_TPU_)

**Figure 4:**
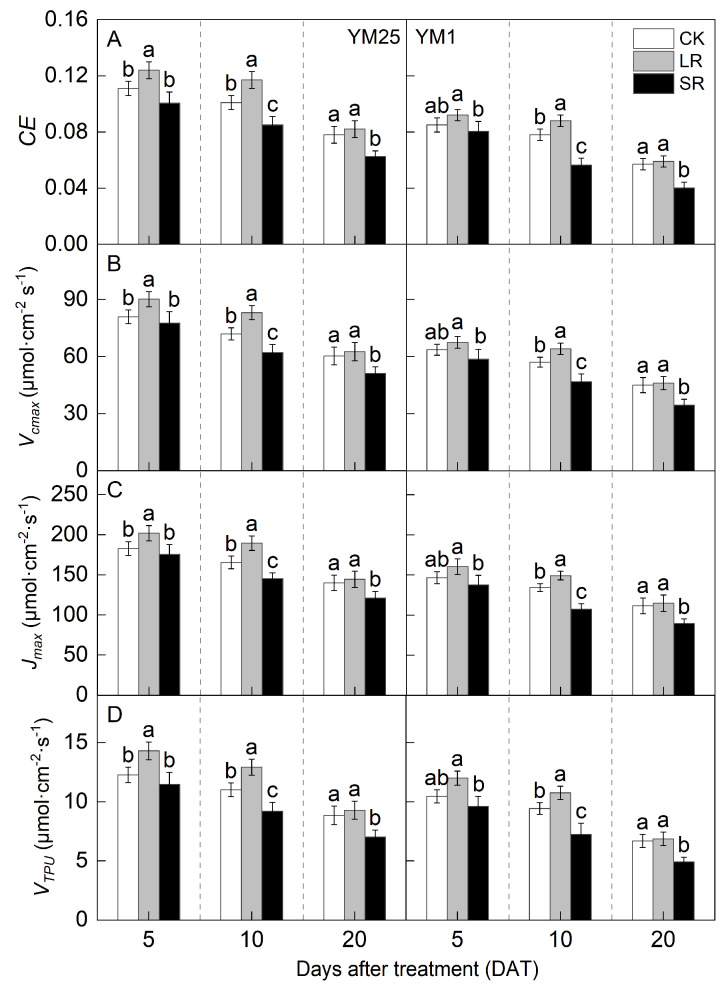
Effects of different source–sink manipulations on carboxylation efficiency (*CE*) (**A**), the Rubisco maximum carboxylation rate (*V_cmax_*) (**B**), the maximum electron transport rate (*J_max_*) (**C**), and the triose phosphate utilization rate (*V_TPU_*) (**D**). CK, control; LR, flag leaf removal; SR, removal of spikelets on one side of each spike. Each value represents the mean ± SD of three replicates. Different lowercase letters indicate statistically significant differences between treatments (*p* < 0.05), according to the LSD test. Error bars indicate SDs.

The *CE*, *V_cmax_*, *J_max_*, and *V_TPU_* values decreased with the number of days after treatment (Figure 4A–D). For LR, the values of *CE*, *V_cmax_*, *J_max_*, and *V_TPU_* in YM25 were significantly higher at 5 and 10 DATs than those of CK. The values of *CE*, *V_cmax_*, *J_max_*, and *V_TPU_* in YM1 were significantly higher at 10 DATs than those of CK. Compared to CK, LR significantly increased the *CE* of YM25 and YM1 by 5.12–15.87% and 2.50–14.12%. LR significantly increased the *V_cmax_* of YM25 and YM1 by 3.89–15.60% and 2.46–12.33%. LR significantly increased the *J_max_* of YM25 and YM1 by 3.09–14.44% and 2.84–11.84%. LR significantly increased the *V_TPU_* of YM25 and YM1 by 4.82–17.37% and 2.62–14.17%. The above results indicate that LR can increase the Rubisco carboxylation capacity, electron transfer rate, and photosynthetic product export capacity of remaining leaves.

For SR, the values of *CE*, *V_cmax_*, *J_max_*, and *V_TPU_* of both cultivars were significantly lower at 10 and 20 DATs than those of CK. Compared to CK, SR significantly decreased the *CE* of YM25 and YM1 by 9.15–19.32% and 5.15–29.43%. SR significantly decreased the *V_cmax_* of YM25 and YM1 by 4.08–15.12% and 7.95–23.30%. SR significantly decreased the *J_max_* of YM25 and YM1 by 3.96–13.38% and 6.25–20.03%. SR significantly decreased the *V_TPU_* of YM25 and YM1 by 6.54–20.63% and 8.01–26.66%. The above results indicate that SR can increase the Rubisco carboxylation capacity, electron transfer rate, and photosynthetic product export capacity in leaves.

### 2.5. Rubisco Activity

**Figure 5:**
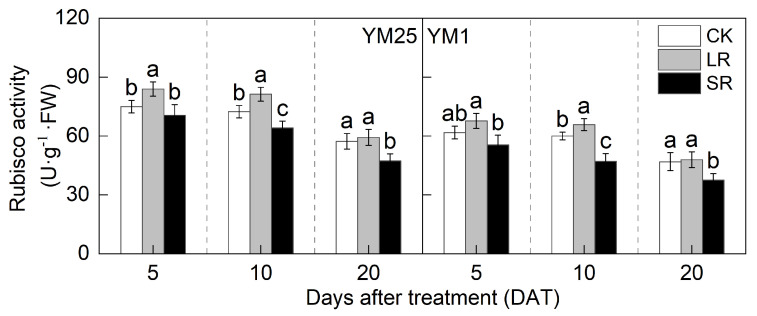
Effects of different source–sink manipulations on Rubisco activity. CK, control; LR, flag leaf removal; SR, removal of spikelets on one side of each spike. Each value represents the mean ± SD of three replicates. Different lowercase letters indicate statistically significant differences between treatments (*p* < 0.05), according to the LSD test. Error bars indicate SDs.

The Rubisco activity decreased with the number of days after treatment (Figure 5). For LR, the Rubisco activity in YM25 was significantly higher at 5 and 10 DATs than that of CK. The Rubisco activity in YM1 was significantly higher at 10 DATs than that of CK. Compared to CK, LR significantly increased the Rubisco activity of YM25 and YM1 by 3.41–12.25% and 2.16–11.25%. For SR, the Rubisco activity of both cultivars was significantly lower at 10 and 20 DATs than that of CK. Compared to CK, SR significantly decreased the Rubisco activity of YM25 and YM1 by 5.96–17.45% and 10.15–21.51%.

### 2.6. Soluble Sugar, Sucrose, and Fructose Contents

**Figure 6:**
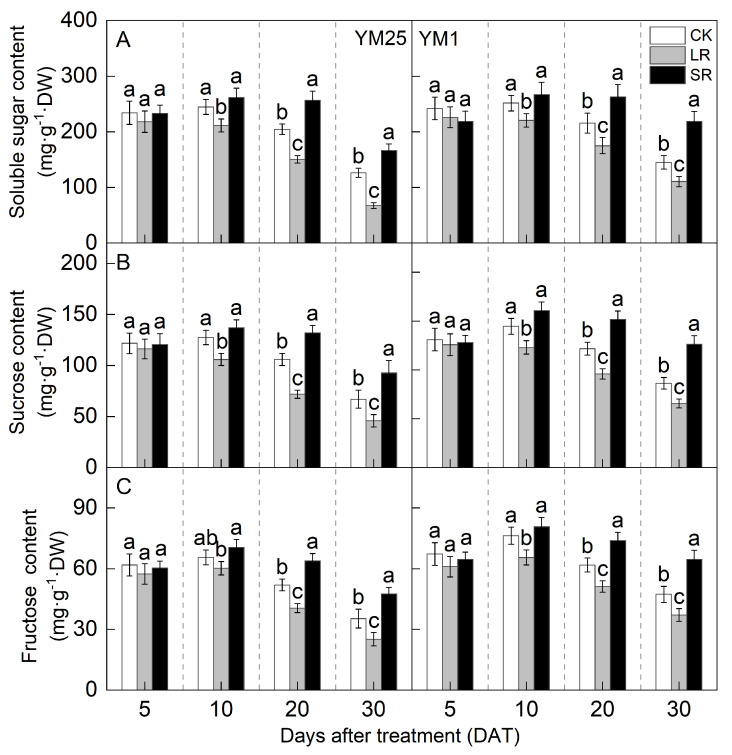
Effects of different source–sink manipulations on soluble sugar contents (**A**), sucrose contents (**B**), and fructose contents (**C**). CK, control; LR, flag leaf removal; SR, removal of spikelets on one side of each spike. Each value represents the mean ± SD of three replicates. Different lowercase letters indicate statistically significant differences between treatments (*p* < 0.05), according to the LSD test. Error bars indicate SDs.

The soluble sugar, sucrose, and fructose contents increased and then decreased with the number of days after treatment and peaked at 10 DATs (Figure 6A–C). For LR, the soluble sugar, sucrose, and fructose contents of both cultivars were significantly lower at 20 and 30 DATs than those of CK. Compared to CK, LR significantly decreased the soluble sugar content of YM25 and YM1 by 6.81–46.63% and 6.64–23.68%. LR significantly decreased the sucrose content of YM25 and YM1 by 4.49–32.17% and 3.95–24.05%. LR significantly decreased the fructose content of YM25 and YM1 by 7.16–28.91% and 9.32–21.51%. The above results indicate that LR can improve the carbohydrate supply capacity of remaining leaves.

For SR, the soluble sugar, sucrose, and fructose contents of both cultivars were significantly higher at 20 and 30 DATs than those of CK. Compared to CK, SR significantly increased the soluble sugar content of YM25 and YM1 by 6.84–31.65% and 5.94–50.74%. SR significantly increased the sucrose content of YM25 and YM1 by 7.45–38.26% and 11.06–46.12%. SR significantly increased the fructose content of YM25 and YM1 by 7.42–34.43% and 5.88–36.56%. The above results indicate that SR can cause carbohydrate accumulation in leaves at the late stage of filling.

### 2.7. Enzyme Activity of Carbohydrate Metabolism

**Figure 7:**
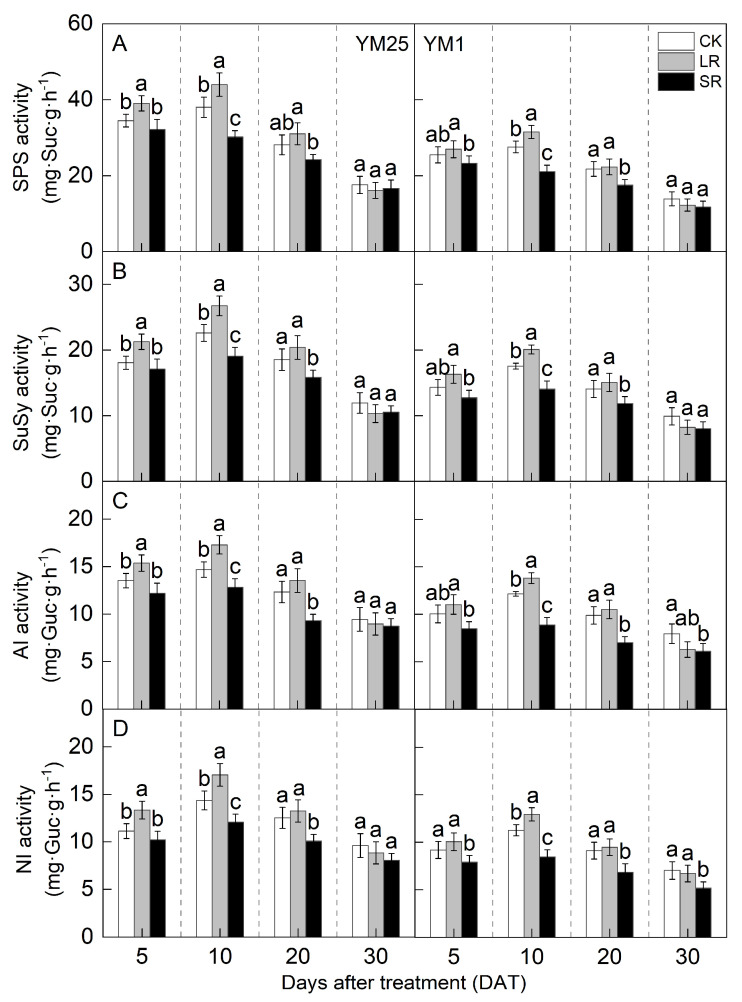
Effects of different source–sink manipulations on sucrose phosphate synthase activity (SPS) (**A**), sucrose synthase activity (SuSy) (**B**), acidic invertase activity (AI) (**C**), and neutral invertase activity (NI) (**D**). CK, control; LR, flag leaf removal; SR, removal of spikelets on one side of each spike. Each value represents the mean ± SD of three replicates. Different lowercase letters indicate statistically significant differences between treatments (*p* < 0.05), according to the LSD test. Error bars indicate SDs.

The SPS, SuSy, AI, and NI activity increased and then decreased with the number of days after treatment and peaked at 10 DATs (Figure 7A–D). For LR, the SPS, SuSy, AI, and NI activity in YM25 was significantly higher at 5 and 10 DATs than that of CK. The SPS, SuSy, AI, and NI activity in YM1 was significantly higher at 10 DATs than that of CK. Compared to CK, LR significantly increased the SPS activity of YM25 and YM1 by 10.28–15.62% and 5.69–12.49%. LR significantly increased the SuSy activity of YM25 and YM1 by 10.10–18.28% and 7.08–14.49%. LR significantly increased the AI activity of YM25 and YM1 by 9.97–17.75% and 6.51–13.70%. LR significantly increased the NI activity of YM25 and YM1 by 5.87–19.95% and 3.96–14.93%. The above results indicate that LR can improve the sucrose synthesis and metabolism of remaining leaves.

For SR, the SPS, SuSy, AI, and NI activity of both cultivars was significantly lower at 10 and 20 DATs than that of CK. Compared to CK, SR significantly decreased the SPS activity of YM25 and YM1 by 5.12–20.59% and 8.63–23.68%. SR significantly decreased the SuSy activity of YM25 and YM1 by 5.43–15.53% and 10.84–19.88%. SR significantly decreased the AI activity of YM25 and YM1 by 7.52–24.28% and 15.58–29.09%. SR significantly decreased the NI activity of YM25 and YM1 by 8.18–19.42% and 14.04–26.72%. The above results indicate that SR can reduce sucrose synthesis and metabolism in leaves.

### 2.8. Relative Expression of SPS1, SUS1, CIN1, and SUT1

**Figure 8:**
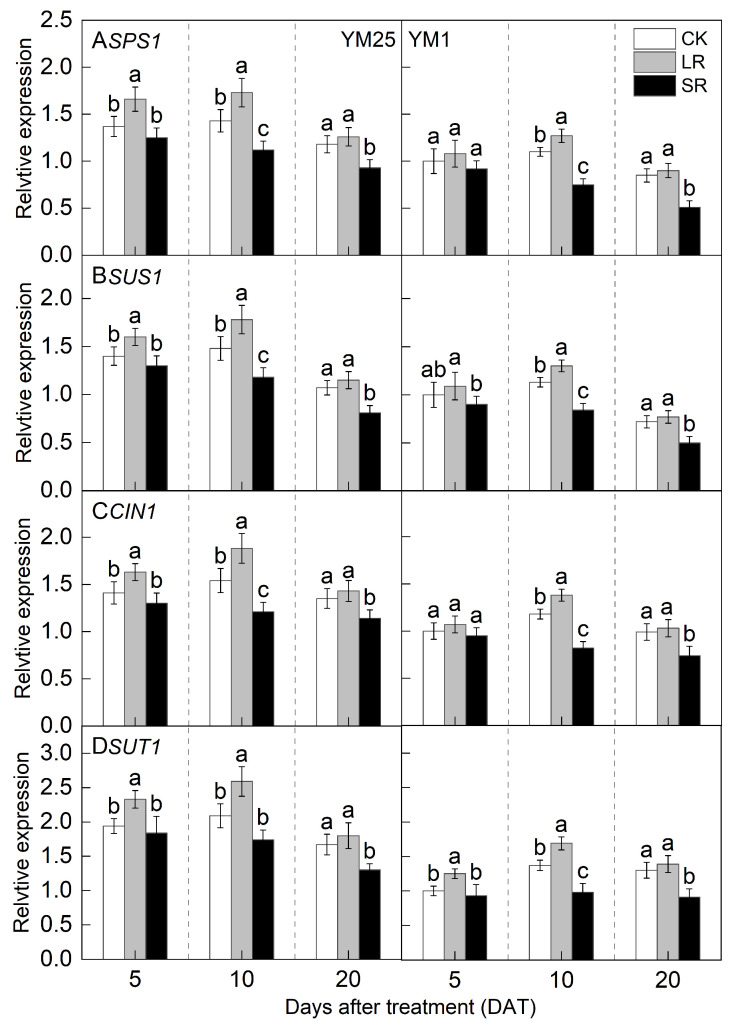
Effects of different source–sink manipulations on the sucrose phosphate synthase gene (*SPS1*) (**A**), the sucrose phosphate synthase gene (*SUS1*) (**B**), the invertase gene (*CIN1*) (**C**), and the sucrose transporter protein gene (*SUT1*) (**D**). CK, control; LR, flag leaf removal; SR, removal of spikelets on one side of each spike. Each value represents the mean ± SD of three replicates. Different lowercase letters indicate statistically significant differences between treatments (*p* < 0.05), according to the LSD test. Error bars indicate SDs.

For LR, the *SPS1*, *SUS1*, and *CIN1* genes in YM25 were significantly up-regulated at 5 and 10 DATs compared with CK (Figure 8A–C). The *SPS1*, *SUS1*, and *CIN1* genes in YM1 were significantly up-regulated at 10 DATs compared with CK. The *SUT1* gene of both cultivars was significantly up-regulated at 5 and 10 DATs compared with CK (Figure 8D). The above results indicate that LR can improve the sucrose transport capacity of remaining leaves.

For SR, the *SPS1*, *SUS1*, *CIN1*, and *SUT1* genes of both cultivars were significantly down-regulated at 10 and 20 DATs compared with CK. The above results indicate that SR can reduce the sucrose transport capacity in leaves.

## 3. Discussion

Leaf removal and spikelet removal alter source–sink relationships during grain filling. Studies have shown that the greatest impact on a yield will be realized through a source–sink manipulation, where an increase in sources only will cause a sink limitation, and an increase in the sink only will lead to a source deficiency [9,12]. Many studies have shown that the removal of both leaves and spikelets during grain filling leads to a significant reduction in grain yields [14,23]. Wang et al. [24] found that a source–sink manipulation significantly affects the accumulation of zinc and other nutrient elements in grains, with the plant hormone ABA promoting grain filling and element distribution, while the ethylene precursor ACC exerts inhibitory effects. It is noteworthy that source–sink manipulation methods, such as partial shading of leaves [21,25] or physical removal [11,14], have been employed in other studies. Shading reduces the source capacity without physical leaf removal, thereby minimizing compensatory effects in remaining tissues. Physical removal (as in our LR/SR treatments) more directly mimics agronomic practices like pest damage or pruning. Despite methodological differences, a consensus exists that a source–sink imbalance universally triggers the feedback regulation of photosynthesis. Flag leaves are generally considered the most important source of photosynthetic substances, closely related to spikelet sterility, thousand-kernel weights, and grain yields [26]. Some studies have reported a decrease in crop yields and thousand-kernel weights due to the removal of flag leaves [27,28]. Their removal not only reduces the total source area but also disrupts the spatial coordination of the assimilate supply that was partially mitigated by compensatory photosynthesis in remaining leaves. In this study, compared with CK, leaf removal (LR) significantly increased the sink–source ratio (23.84%) and decreased the yield (16.17%), 1000-kernel weight (11.73%), and kernels per spike (7.33%) of two cultivars (Table 1). These results are consistent with previous findings [28]. Compensation or reactivation in remaining photosynthetic organs may occur when the photosynthetic organs of a plant are separated [29]. Even so, the 1000-kernel weight and yield were still reduced by LR (11.73% and 16.17%), which means that the functional leaves, especially the flag leaves, were the most important source for photosynthetic production [26]. This finding supports previous studies that have reported that flag leaf photosynthesis contributes an average of 5–15% of the final grain yield under irrigated conditions [30]. Several previous studies have found that spikes contribute more to grain yields than leaf photosynthesis, and one way to increase wheat yields is to manipulate the sink (grain) capacity [18]. Other studies have reported that the removal of spikelets increased the grain weight and the number of remaining grains in wheat [31]. In this study, compared with CK, spikelet removal (SR) significantly decreased the sink–source ratio (44.12%) and increased the 1000-kernel weight (10.02%) of the remaining grains but decreased the yield (43.93%) and kernels per spike (49.31%) of two cultivars (Table 1). This study showed that the removal of half of the spikelets resulted in a 43.93% decrease in the yield and a 10.02% increase in the 1000-kernel weight. The decrease in the grain yield by SR was less than the number of kernels per spike, which was due to the compensatory increase in the single grain weight.

Source–sink relationships are an intrinsic factor affecting crop photosynthesis [7]. Source–sink manipulations cause changes in source–sink structures, resulting in a reduction or accumulation of photosynthetic products in the leaves, which affects photosynthesis and the transport and distribution of photosynthetic products between source and sink organs [32]. LR can influence the growth and photosynthetic capacity of plants, and it remobilizes carbon and nitrogen reserves [33]. Studies have shown that an appropriate reduction in source leaves results in enhanced light transmission performance of the crop population and enhances the activity of the sink organs and transport tissues, and the photosynthetic capacity of the remaining leaves increases [34]. However, an excessive reduction in source leaves will cause the leaf population’s photosynthesis and photosynthetic products to decrease, and the yield will be reduced instead [35]. In this study, compared with CK, LR significantly increased the SPAD (4.05–11.67%) (Figure 1) and Pn (4.27–15.82%) (Figure 2) of the remaining leaves in the short term, indicating that a photocomposition effect might occur. This result is consistent with previous findings [11]. SPAD dynamic trends were closely aligned with the observed changes in Pn, demonstrating that source–sink manipulations significantly influenced the leaf physiological activity and senescence progression. LR significantly increased Φ_PSII_ (2.98–15.22%) and ETR (3.97–14.93%) (Figure 3). This suggests that removing leaves improved the actual photochemical efficiency and electron transfer capacity. In addition, LR significantly increased the *CE*, *V_cmax_*, and Rubisco activity (2.16–12.25%) (Figure 4 and Figure 5). This suggests that removing leaves improved the Rubisco carboxylation capacity, which improved the photosynthetic capacity of the remaining leaves. The sink–source ratio is also an important factor influencing the allocation of assimilates, regulating the functioning of wheat grain growth stages. Studies have shown that removing leaves significantly increases the activity of remaining leaf sources and improves the translocation of assimilates to the grain sink [34]. In this study, LR significantly increased the activities of enzymes related to sucrose metabolism (SPS, SuSy, AI, and NI) (3.96–19.95%) and gene expression (*SPS1*, *SUS1*, *CIN1*, and *SUT1*) of the remaining leaves (Figure 7 and Figure 8), which accelerated the transport of soluble sugar, sucrose, and fructose (Figure 6). These results suggest that the removal of leaves improves the sucrose synthesis and transport capacity of remaining leaves, facilitates the adequate transport of photosynthetic products to the grain sink, stimulates the source activity to a certain extent, and ultimately improves the photosynthetic capacity of the source leaves.

Studies have shown that the removal of spikelets inhibits sucrose export by reducing the sink strength, leading to the accumulation of photosynthetic products in leaves; decreased leaf Rubisco enzyme activity; reduced PSII efficiency; and, ultimately, reduced Pn [28]. In this study, compared with CK, SR reduced the SPAD (5.50–20.86%) (Figure 1) and Pn (8.54–21.41%) (Figure 2) of the flag leaves. This was attributed to the removal of spikelets, reducing the sink size and the demand for photosynthetic products and reducing the photosynthetic ability of the source leaves. SR accelerated chlorophyll degradation and reduced Pn, consistent with the feedback inhibition of photosynthesis under sink limitations. SR significantly reduced Φ_PSII_ (7.12–17.86%) and ETR (3.51–16.71%) (Figure 3). This suggests that removing spikelets limited the actual photochemical efficiency and electron transfer capacity. In addition, SR significantly reduced the *CE*, *V_cmax_*, and Rubisco activity (5.96–21.51%) (Figure 4 and Figure 5). This suggests that removing spikelets reduced the Rubisco carboxylation capacity, leading to a decrease in the leaf photosynthetic capacity. It has been shown that removing spikelets inhibits the transport from the source end to the sink organs, and the blockage of sucrose export causes the accumulation of end-products in the source leaves, which often leads to a decrease in the photosynthetic efficiency of the source leaves [14]. Since sucrose transport is mainly carried out by the phloem, SR may result in higher sucrose concentrations in the phloem of leaves [36]. In this study, SR significantly reduced the activities of enzymes related to sucrose metabolism (SPS, SuSy, AI, and NI) (5.12–29.09%) and gene expression (*SPS1*, *SUS1*, *CIN1*, and *SUT1*) in the flag leaves (Figure 7 and Figure 8), and soluble sugars, sucrose, and fructose were accumulated in the leaves (Figure 6). These results suggest that the removal of spikelets results in the accumulation of photosynthetic products in the source leaves, producing feedback inhibition, which leads to a decrease in sucrose synthesis and the transport capacity, which in turn reduces the photosynthetic capacity. In summary, the increase in the photosynthetic capacity by a higher sink–source ratio was mainly due to the light compensation effect of the remaining leaves, and the accelerated transport of assimilates further improved photosynthesis but did not compensate for the negative effect of the leaf source deficit. The decrease in the photosynthetic capacity with a lower sink–source ratio was mainly due to feedback inhibition of photosynthetic products, where sucrose accumulation and assimilate translocation in the source leaves were impeded, inhibiting photosynthesis. A higher sink–source ratio is an important indicator of high photosynthetic efficiency, which can be used as a screening and judgment index in variety selection and cultivation regulation.

In addition, the observed sensitivity of source-limited wheat to an increased sink–source ratio may be related to dynamic adjustments in sucrose transport and carbon partitioning at the molecular level. Specifically, the up-regulation of *SUT1* expression under LR treatments likely enhances phloem loading efficiency, facilitating rapid sucrose export from the source leaves to the sink organs (Figure 8). This process alleviates carbohydrate accumulation in leaves, thereby mitigating feedback inhibition of photosynthesis [37]. In source-limited wheat, where the sink demand exceeds the source capacity, *SUT1*-mediated sucrose efflux becomes critical to maintaining photosynthetic efficiency by preventing excessive sucrose accumulation. Conversely, in sink-limited wheat, the reduced *SUT1* expression under SR treatments exacerbates leaf sucrose accumulation (Figure 6), triggering the down-regulation of Rubisco activity and PSII efficiency (Figure 4 and Figure 5) through mechanisms involving trehalose-6-phosphate signaling, a key regulator of source–sink crosstalk. The different responses of source-limited versus sink-limited wheat highlight the importance of *SUT1* as a molecular rheostat-balancing source activity and sink strength, offering a potential target for breeding wheat varieties with optimized source–sink coordination under fluctuating environmental or agronomic conditions.

## 4. Materials and Methods

### 4.1. Experiment Design

**Figure 9:**
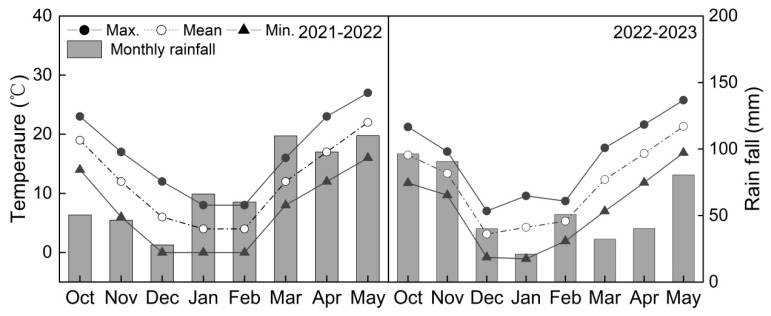
Monthly maximum (Max.), minimum (Min.), and mean temperatures and monthly rainfall for the two wheat-growing seasons (2021–2022 and 2022–2023).

The experiment was conducted at the experimental base of Nanjing Agricultural University (32°24′ N, 118°9′ E) in Nanjing, China, during the 2021/2022 and 2022/2023 growing seasons. The soil at the experimental site was clay loam. Soil samples at a depth of 0 to 25 cm were found to have 16.98 g kg^−1^ of organic matter, 1.40 g kg^−1^ of total N content, 68.22 mg kg^−1^ of available N content, 12.37 mg kg^−1^ of available P_2_O_5_ content, and 93.76 mg kg^−1^ of rapidly available K in 2021–2022 and 18.75 g kg^−1^ of organic matter, 1.59 g kg^−1^ of total N content, 71.34 mg kg^−1^ of available N content, 16.86 mg kg^−1^ of available P_2_O_5_ content, and 112.76 mg kg^−1^ of rapidly available K in 2022–2023. A completely randomized experimental design with three replicates was used. Two wheat cultivars with different source–sink relationships were selected. Yangmai 1 (YM1) is a sink-limited cultivar, and Yangmai 25 (YM25) is a source-limited cultivar, which were studied in our previous work [38]. YM25 is a compact plant type with a large-spiked; large-grained; dwarf-stem; and a long, broad-shaped leaf. YM1 is a loose plant type with a small-spiked; small-grained; high-stem; and a short, narrow leaf. According to the response of different cultivars to source and sink manipulations, YM25 has a higher sink–source ratio, manifesting as a source-limited cultivar. YM1 has a lower sink–source ratio, manifesting as a sink-limited cultivar. Then, an additional 240 kg ha^−1^ of N was set. The basic seeding was 2.25 × 10^6^ seeds ha^−1^. The plot size was 3.0 m × 3.0 m (9 m^2^), with a spacing of 0.25 m between rows (12 rows per plot). The N fertilizers were applied at a sowing-and-jointing ratio of 1:1, together with 150 kg of P_2_O_5_ ha^−1^ and 150 kg of K_2_O ha^−1^. In addition, leaves and spikelets were removed from selected plants at anthesis for source and sink manipulations. In each sampling plot, three treatments were conducted in three subplots, i.e., (1) all flag leaves were removed at anthesis using scissors in subplot 1 to reduce the utilization of source assimilates (LRs); (2) the spikelets on one side of each spike were removed at anthesis using tweezers in subplot 2 to double the assimilated utilization of the remaining grains (SRs); and (3) subplot 3 was set for a control (CK), respectively. Each subplot consisted of 4 rows that were 3 m long. The area of each microzone was 1 m × 3 m. The seeding and harvesting dates of the wheat in each ecological site were accurately recorded, with sowing dates of 13 November 2021 and 29 October 2022 and harvesting dates of 26 May 2022 and 29 May 2023. Managed according to local cultivation measures, insecticides and fungicides were used as needed to control pests and diseases. Single stems of wheat with uniform growth and flowering on the same day were selected for tagging at anthesis. The average temperature and cumulative precipitation meteorological conditions during wheat growth were calculated according to the meteorological data provided by the local weather station and are shown in Figure 9. The annual rainfall was 568.76 and 452.7 mm during 2021–2022 and 2022–2023, respectively. The range of average temperatures in the region was 4–22 °C and 3–21 °C during 2021–2022 and 2022–2023, respectively.

### 4.2. Yield and Its Components and Sink–Source Ratio

The spike number per m^2^ was recorded at physiological maturity when the plants were cut at the soil level with a sickle, harvested, threshed, dried, and weighed by hand to provide the grain yield (kg·ha^−1^) and 1000-kernel weight, with the grain moisture adjusted to 13%. Thirty spikes were selected randomly in the middle of each plot to measure the kernels per spike. The leaf area was used as the source, and the grain size (grain weight) and number were used as the sink. The sink–source ratio can be used as an indicator to reflect source–sink relationships. The sink–source ratio was calculated from the single spike yield at maturity divided by the leaf area per culm at anthesis.

### 4.3. SPAD, Pn, and Chlorophyll Fluorescence Measurement

The chlorophyll SPAD of the leaves was measured at 5, 10, 20, and 30 days after treatment (DATs). The SPAD-502 chlorophyll meter (Konica Minolta Holdings, Inc., Tokyo, Japan) was used to directly measure the SPAD value of the wheat leaf chlorophyll in the field. A total of 6 leaves were randomly measured in each community, with the measurement locations uniformly located at the upper, middle, and lower positions of each leaf. They were measured three times to obtain the average value.

The Pn of the leaves was measured by the gas exchange system (LI-6400, LI-Cor Inc., Lincoln, NE, USA) during 09:00–11:00 a.m. on clear days without clouds at 5, 10, 20, and 30 days after treatment (DATs). The environmental conditions in the leaf chamber were according to Mu et al. [39]. The photosynthetic active radiation (PAR) was 1200 μmol·m^−2^·s^−1^, the leaf chamber CO_2_ concentration was about 400 ± 20 μmol·CO_2_·L^−1^, the leaf temperature was 25.0 ± 0.5 °C, and the humidity was 60%. Three leaves of uniform growth were selected from each plot, and the Pn was recorded.

The chlorophyll fluorescence of the leaves was measured at 5, 10, and 20 DATs by using the chlorophyll fluorometer PAM-2500 (WALZ Inc., Effeltrich, Germany), noted as Φ_PSII_, F_v_/F_m_, F_o’_, F_o_, F_s_, F_m’_, and F_m_. Φ_PSII_, F_v_/F_m_, QL, andETR were calculated. Chlorophyll fluorescence was measured in parallel with the Pn. The chlorophyll fluorescence parameters were determined using a kinetic imaging fluorometer, following the method described by Shao et al. [40]. Φ_PSII_ = (F_m’_ − F_s_)/F_m_^’^, F_v_/F_m_ = (F_m_ − F_o_)/F_m_ [41], QL = F_o’_/F_s_ × (F_m_ − F_s_)/(F_m’_ − F_o’_) [42], and ETR = (F_m’_ − F_s_)/F_m’_ × PPFD × 0.85 × 0.5 [43].

### 4.4. CE, V_cmax_, J_max_, and V_TPU_

The Pn/intercellular CO_2_ concentration (A–C_i_) curve was measured by an LI-6400 (LI-Cor Inc., Lincoln, NE, USA) portable gas exchange measurement system with a red and blue light source. The photosynthetic active radiation (PAR) was 1200 μmol·m^−2^·s^−1^, the leaf chamber CO_2_ concentration was about 400 ± 20 μmol·CO_2_·L^−1^, the leaf temperature was 25.0 ± 0.5 °C, and the humidity was 60%. Measurements were performed in cloud-free weather during 09:00–11:00 a.m. on clear days without clouds. Leaves were placed in a leaf chamber with a reference CO_2_ concentration of 400 μmol·CO_2_·L^−1^ for at least 10 min before measurement. The reference CO_2_ concentration was 400, 200, 150, 100, 50, 400, 600, 600, 800, 1000, 1200, 1600, and 1800 μmol·CO_2_·L^−1^, and data were recorded after CO_2_ reached a steady state (2–3 min). Plotting A–C_i_ curves, the initial slope of the curve represents *CE* when Ci was <200 μmol·CO_2_·L^−1^ [43]. The *V_cmax_*, *J_max_*, and *V_TPU_* were calculated according to modified equations [44]. The A–Ci curve is shown in Supplementary Figure S1.

### 4.5. Rubisco Activity

Frozen samples were taken in each plot at 5, 10, and 20 DATs. All samples were frozen immediately in liquid nitrogen and stored in a −80 °C environment. The total Rubisco activity was determined according to Zheng et al. [45]. A total of 100 μL of crude extract was activated with 100 μL of an activation solution for 10 min at 25 °C. A rapid assay of 100 μL of crude extract was performed with 700 μL of an assayed buffer and 200 μL of 10 mM of RuBP. Changes in absorbance were monitored at 340 nm for 60 s (10 s per measurement).

### 4.6. Soluble Sugar, Sucrose, and Fructose Contents

Samples were taken in each plot at 5, 10, 20, and 30 DATs. Soluble sugar, sucrose, and fructose were extracted from the plant materials by using an 80% ethanol solution. The soluble sugar content was determined by the anthrone method [46]. The sucrose and fructose contents were measured using the resorcinol method [47].

### 4.7. Enzyme Activity of Carbohydrate Metabolism

Frozen samples were taken in each plot at 5, 10, 20, and 30 DATs. All samples were frozen immediately in liquid nitrogen and stored in a −80 °C environment. Frozen samples were used to measure the activities of enzymes, including sucrose phosphate synthase (SPS) and sucrose synthetase (SuSy), according to the method of Liu et al. [30]. Acidic/neutral invertase activity (AI/NI) was extracted as described by Miron and Schaffer [48].

### 4.8. qRT-PCR Analysis

The total RNA was extracted from leaves using RNAiso Plus (Takara, Dalian, China), according to the manufacturer’s instructions. qRT-PCR was carried out as described by Ren et al. [49]. Primers were designed using the Primer Premier 5.0 software (Premier Biosoft International, Palo Alto, CA, USA). The reference sequence was *TaADP-RF* (ADP-ribosylation factor). The reference genes and the related gene primer sequences are shown in Supplementary Table S1 in our previous work [38]. Each treatment consisted of three biological replicates. The obtained data were used to calculate the relative gene expressions using the 2^−ΔΔCT^ method [50].

### 4.9. Statistical Analysis

A statistical analysis was performed using SPSS 19.0 (SPSS Inc., Chicago, IL, USA) and included a one-way and a two-way analysis of variance (ANOVA). For presenting the data in charts, Duncan’s test was conducted, with statistical significance accepted at *p* < 0.05 (LSD_0.05_). All presented graphs were produced using Origin 2021 (OriginLab, Northampton, MA, USA). Each value represents the mean ± SD of three replicates.

## 5. Conclusions

Increasing the sink–source ratio significantly improves the photosynthetic characteristics of wheat. Compared with CK, leaf removal (LR) increased the sink–source ratio (23.84%) and significantly reduced the yield (16.17%), 1000-kernel weight (11.73%), and kernels per spike (7.33%). LR increased the leaves’ Pn (4.27–15.82%), the electron transfer rate (3.97–14.93%), and the Rubisco activity (2.16–12.25%) in the short term. LR increased sucrose synthesis-related enzyme activities (3.96–19.95%) and gene expressions *(SPS1*, *SUS1*, *CIN1*, and *SUT1*). Compared with CK, spikelet removal (SR) reduced the sink–source ratio (44.12%) and significantly increased the 1000-kernel weight (10.02%) but reduced the yield (43.93%) and kernels per spike (49.31%). SR reduced the leaves’ Pn (8.54–21.41%), the electron transfer rate (3.51–16.71%), and the Rubisco activity (5.96–21.51%). SR decreased sucrose synthesis-related enzyme activities (5.12–29.09%) and gene expressions *(SPS1*, *SUS1*, *CIN1*, and *SUT1*). Therefore, a higher sink–source ratio is an important indicator of high photosynthetic efficiency, which can be used as a screening and judgment index in variety selection and cultivation regulation.

## Figures and Tables

**Table 1:** Combined analysis of variance for yield components and sink–source ratio under different source–sink manipulations for the two wheat-growing seasons (2021–2022 and 2022–2023).

Cultivars	Treatment	Grain Yield (kg·ha^−1^)	Kernels Per Spike	1000-Kernel Weight (g)	Leaf Area (cm^2^)	Sink–Source Ratio (mg·cm^−2^)
2021–2022						
YM25	CK	8581 a	44.55 a	44.52 b	88.68 a	22.36 b
	LR	7317 b	42.56 b	38.92 d	58.44 c	28.35 a
	SR	5125 c	23.87 e	49.75 a	90.54 a	13.12 d
YM1	CK	6967 b	36.01 c	38.41 d	68.96 b	20.06 c
	LR	5522 c	33.05 d	32.48 e	45.71 d	23.48 b
	SR	3646 d	17.54 f	41.48 c	70.28 b	10.35 e
F-value	F_C_	224.28 **	331.25 **	181.25 **	76.73 **	40.49 **
	F_T_	327.77 **	759.29 **	124.37 **	83.98 **	257.19 **
	F_C×T_	0.71	4.45 *	1.70	1.46	2.28
2022–2023						
YM25	CK	9292 a	46.90 a	42.76 b	90.03 a	22.28 b
	LR	8072 b	43.40 b	39.66 c	58.54 c	29.40 a
	SR	5460 d	23.53 e	48.97 a	87.01 a	13.24 d
YM1	CK	7792 b	40.12 c	37.58 d	76.73 b	19.65 c
	LR	6308 c	36.44 d	33.2 e	51.54 d	23.48 b
	SR	4165 e	20.18 f	40.03 c	76.40 b	10.57 e
F-value	F_C_	160.28 **	84.48 **	182.53 **	24.64 **	51.12 **
	F_T_	329.89 **	467.21 **	84.33 **	78.26 **	262.43 **
	F_C×T_	1.28	3.57	4.75 *	0.77	4.34 *

CK, control; LR, flag leaf removal; SR, removal of spikelets on one side of each spike. Values are means of three replicates. F-values were calculated by two-way ANOVA, * represents *p* < 0.05, ** represents *p* < 0.01, and different letters in a column indicate significant differences (*p* < 0.05), according to the LSD test.

## Data Availability

The original contributions presented in this study are included in the article/Supplementary Materials. Further inquiries can be directed to the corresponding authors.

## Supplementary Materials

The following supporting information can be downloaded at: https://www.mdpi.com/article/10.3390/plants14101456/s1.
