# Supplementary material for: Regulatory Effects of Source–Sink Manipulations on Photosynthesis in Wheat with Different Source–Sink Relationships
Source: Plants (Basel). 2025 May 13;14(10):1456. doi: 10.3390/plants14101456 (PMC12115351; doi:10.3390/plants14101456)
Supplement: Supplementary file 1 [file plants-14-01456-s001.zip › plants-3610518-supplementary.pdf]

Regulatory Effects of Source–Sink Manipulations on Photosynthesis in Wheat with Different Source–Sink Relationships

Supplementary Table S1 Primer sequences used for qRT-PCR expression analysis. *SPS1*, sucrose phosphate synthase gene; *SUS1*, sucrose phosphate synthase gene; *CIN1*, invertase gene; *SUT1*, sucrose transporter protein gene.

Genes	Primer sequence (5'-3')	
	Forward primer	Reverse primer
SPS1	ACCTGTTTACTCGCCAAGT	TCCCTCAATACGCCTCAT
SUS1	TGACTATGATGCTGAACGA	AGGACCTTCTCAAGACGCT
CIN1	ATCCAGCCCAGCATCAAGT	TAGGTGCCGACGGTGTAGT
SUT1	TGGATTCTGGCTCCTTGAC	GCCATCCAAGAACAGAAGATT
ADP-RF	GCTCTCCAACAACATTGCCAAC	GCTTCTGCCTGTCACATACGC

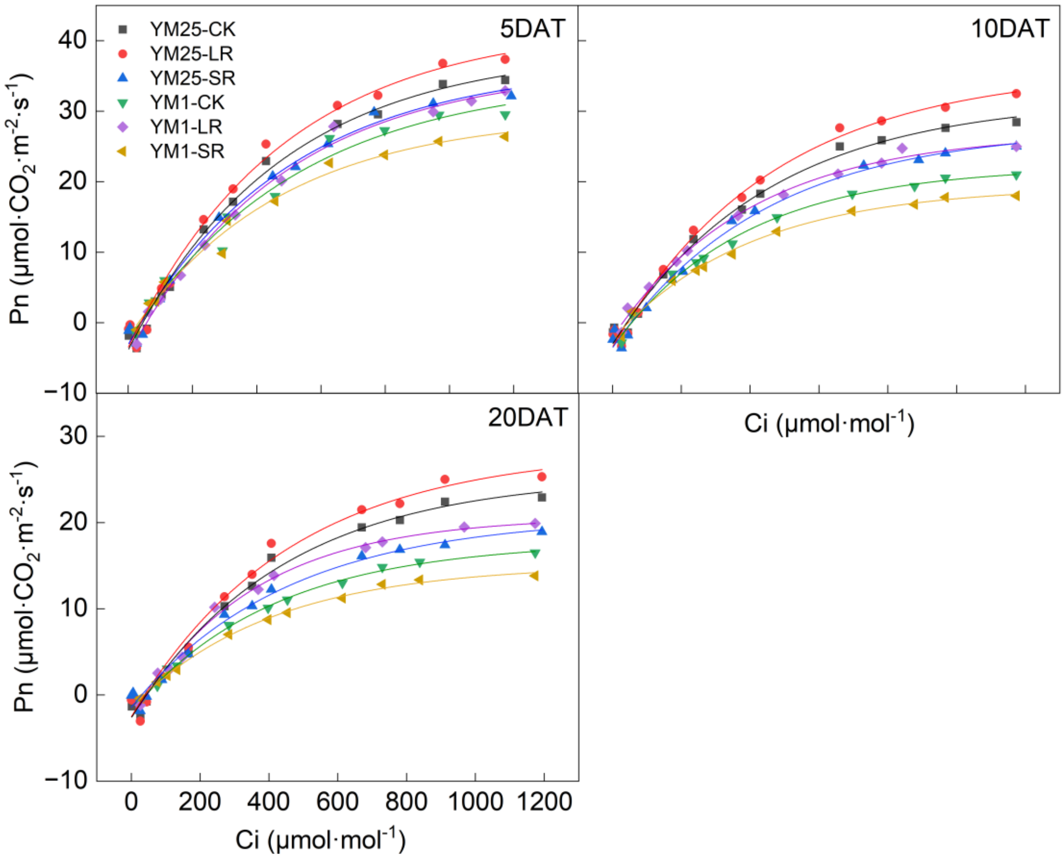

Supplementary Figure S1 The A-Ci curve of different source-sink manipulations. CK, control; LR, removal of flag leaves; SR, removal of the spikelets on one side of each spike.
